# Increased risk of revision of acetabular cups coated with hydroxyapatite: A Swedish Hip Arthroplasty Register study involving 8,043 total hip replacements

**DOI:** 10.3109/17453670903413178

**Published:** 2010-03-31

**Authors:** Stergios Lazarinis, Johan Kärrholm, Nils P Hailer

**Affiliations:** ^1^Department of Orthopaedics, Institute of Surgical Sciences, Uppsala University HospitalUppsala; ^2^Department of Orthopaedics, Institute of Surgical Science, Sahlgrenska University Hospital, Göteborg University, MölndalSweden

## Abstract

**Background:**

Hydroxyapatite (HA) is the main inorganic component of bone, and HA coating is widely used on acetabular cups in hip arthroplasty. It has been suggested that this surface finish improves cup survival.

**Methods:**

All patients registered in the Swedish Hip Arthroplasty Register between 1992 and 2007 with an uncemented acetabular implant that was available either with or without HA coating were identified. 8,043 total hip arthroplasties (THAs) with the most common cup types (Harris-Galante, Romanus, and Trilogy) were investigated. A Cox regression model including type of coating, age, sex, primary diagnosis, cup type, and type of stem fixation was used to calculate adjusted risk ratios (RRs) for the risk of revision.

**Results:**

HA coating was a risk factor for cup revision due to aseptic loosening (adjusted RR 1.7; 95% CI: 1.3–2). Age at primary arthroplasty of < 50 years, a diagnosis of pediatric hip disease, the use of a cemented stem, and the Romanus and Harris-Galante cup types were also associated with statistically significantly increased risk of cup revision due to aseptic loosening.

**Interpretation:**

Our findings question the routine use of HA-coated cups in primary total hip arthroplasty. With some designs, this practice may even increase the risk of loosening—resulting in revision surgery.

## Introduction

Hydroxyapatite (HA) is the main inorganic component of human bone. It was therefore hypothesized early on that coating of metallic implants with HA would enhance bone ingrowth and thus lead to better initial or even long-term stability. There was experimental evidence that HA was in fact well tolerated by the organism without leading to foreign body reactions (Nery et al. 1975), that HA coating enhanced bone ingrowth (Jarcho et al. 1977), and that implants coated with HA showed improved fixation to surrounding bone tissue (Ducheyne et al. 1980).

Based on these findings, HA coating was applied to acetabular and femoral arthroplasty components. Initial reports on acetabular cups coated with HA suggested reduced rotational cup migration and reduced incidence of radiolucent lines (Moilanen et al. 1996), with few revisions after short- or medium-term follow-up (Roffman and Kligman 1999, Ali and Kumar 2003). On the other hand, several publications on cohorts of less than 200 patients described high failure rates of various cups coated with HA (Reikeras and Gunderson 2002, Lai et al. 2002, Miyakawa et al. 2004, Cheung et al. 2005, Kim et al. 2006), sometimes due to severe osteolysis and third-body wear secondary to abrasion of HA particles (Morscher et al. 1998).

Despite the fact that it is unclear whether HA coating is beneficial to acetabular implant survival, the orthopedic implant industry nowadays provides a vast array of cups and stems that have been coated with HA. Moreover, these implants are more expensive than those without such coating. Thus, the question of whether HA coating is beneficial is both medically and economically relevant.

We analyzed patient data on uncemented cups with or without HA coating that had been recorded in the Swedish Hip Arthroplasty Register. Our hypothesis was that coating of acetabular cups with HA would influence the risk of cup revision due to aseptic loosening and osteolysis. Our primary endpoint was revision due to aseptic loosening, which in the Swedish Hip Arthroplasty register also includes osteolysis. Secondary endpoints were cup revision for any reason, and cup revision due to infection.

## Patients and methods

### Sources of data

Data were extracted from the Swedish Hip Arthroplasty Register. Every Swedish citizen has a personal identification (social security) number that is linked to information on all changes relevant to the follow-up, such as change of address, date of emigration, or the date of death. All orthopedic units in Sweden that perform total hip arthroplasty, both public and private, report to the register. Revisions have been registered since 1979. All reoperations (any secondary operation of the hip) and revisions (exchange or removal of any of the components) are continuously reported by all operating units in Sweden. The Swedish Hip Arthroplasty Register has been repeatedly validated (Söderman et al. 2000, 2001).

Since 1979, over 270,000 primary total hip replacements and 32,000 revisions have been recorded. Until 1991, data were aggregated in terms of hospital and were not linked to the personal ID number. In 1992 this was changed, which enabled more detailed and reliable studies of individual implant designs, as in this study. This recording also included whether or not there was HA coating. Some frequently used cups initially only available without HA coating were later modified to also embrace a version with HA coating added on to the surface of the original cup. This enables comparative retrospective analyses of components with identical design apart from their surface finish.

### Study population

All primary total hip arthroplasties (THAs) registered in the Swedish Hip Arthroplasty Register between 1992 and 2007 using an uncemented cup that was available with or without HA coating were identified (8,705 hips). There were 5 such cup designs: Trilogy (n = 5,536 hips), Romanus (n = 1,531), Harris-Galante II (n = 976), Reflection (n = 437), and Biomex (n = 225). To reduce the risk of bias caused by patient selection, surgical preference and technique, and other factors not recorded in the register, only acetabular implants with 500 or more registered hips per implant were included (Trilogy, Romanus, and Harris-Galante). This left a study population of 6,646 patients with 8,043 THAs (Tables 1 and 2).

**Table 1.** Characteristics of the patients studied (n = 6,646) from the Swedish Hip Arthroplasty Register (1992–2007)

	n	%
Sex		
Male	3,367	51
Female	3,279	49
Age (years)		
0–49	1,705	26
50–59	2,614	39
60–75	2,142	32
> 75	185	3
Primary diagnosis		
Primary OA	5,037	77
Inflammatory disease	324	5
Fracture	257	4
Pediatric hip disease	599	9
Idiopathic femoral head necrosis	210	3
Secondary posttraumatic OA	37	0.6
Tumor	7	0.1
Other	50	0.8
Total	6,646	100

**Table 2.** Acetabular components in the hips studied

	+ HA		– HA		Total
	n	%	n	%	
Trilogy	4,501	86	1,035	37	5,536
Romanus	440	9	1,091	38	1,531
Harris-Galante	272	5	704	25	976
Total	5,213	100	2,830	100	8,043

The Trilogy (Zimmer Inc., Warsaw, IN) and Harris-Galante II (also Zimmer) cups are hemispherical press-fit shells. The Romanus cup is hemispherical and threaded (Biomet, Warsaw, IN). All 3 designs are made of titanium alloy supplied with a porous coating made of commercially pure titanium. All implants were available with or without HA coating. The ceramic coating on the Harris-Galante II and Trilogy cups consists of a mixture of HA (70%) and tricalcium phosphate (TCP, 30%), whereas the Romanus cup is coated with 60% HA and 40% TCP. Different types of cemented and uncemented stems were combined with these cups, creating hybrid and totally uncemented systems (Table 3). The material of the femoral heads was not recorded and there was no reliable recording of head diameter, which precluded any statistical analysis of these parameters.

**Table 3.** Distribution of cemented and uncemented stems

Uncemented cups:	+ HA		– HA		Total	
	n	%	n	%	n	%
Cemented stem	2,919	56	1,495	53	4,414	55
Uncemented stem	2,287	44	1,331	47	3,618	45

### Statistics

Follow-up started on the day of primary THA and ended on the day of revision, death, emigration, or December 31, 2007, whichever came first.

A Cox proportional hazards model was applied in order to analyze the relative risk (RR) of revision (with 95% confidence intervals (CIs)) due to aseptic loosening, revision due to infection, or for any reason, mutually adjusted for relevant covariates: absence or presence of HA coating, sex, age (< 50, 50–59, 60–75, > 75 years), primary diagnosis before arthroplasty (primary osteoarthritis (OA), inflammatory disease (e.g. rheumatoid arthritis, morbus Bechterew), femoral neck fracture, pediatric hip disease, idiopathic femoral head necrosis, secondary posttraumatic OA, tumor, and other diagnoses), cup design (Harris-Galante, Romanus, Trilogy), and type of stem fixation (cemented or uncemented). The assumption of proportional hazards was investigated by hazard function plots and log-log plots of all covariates. No sign of insufficient proportionality was detected in the hazard functions, and log-log plots ran strictly parallel for all covariates. It has been pointed out that the inclusion of both joints in bilaterally operated patients can create dependency problems (Ranstam 2002), and we therefore investigated whether our Cox regression model was robust against this potential violation. Separate analyses were made, either after including all joints (8,043 hips in 6,646 individuals) or after excluding the second side in bilaterally operated patients (6,646 hips in 6,646 individuals). The calculated crude and adjusted risk ratios were not affected by the inclusion of both THAs of bilaterally operated patients (supplementary Tables 4 and 5 describe the results after exclusion of the second side in bilaterally operated patients; see supplementary data). In addition, in a separate analysis the variable “first or second operated side” was entered as a time-dependent covariate in a Cox regression model including all other covariates mentioned above. Again, this procedure did not affect the results. Moreover, previous authors have shown that inclusion of both sides in bilaterally operated patients in Cox regression models is feasible (Lie et al. 2004, Thillemann et al. 2008). We therefore included all 8,043 THA in our study.

**Table 4.** Relative risk (RR) of cup revision due to aseptic loosening. 8,043 hips in 6,646 patients.

Endpoint: aseptic loosening	No. of patients	No. of revisions	Crude RR (95% CI)	Adjusted RR (95% CI)
Coating				
– HA	2,830	257	1.0 (ref) ^a^	1.0 (ref)
+ HA	5,213	180	1.12 (0.98–1.46)	1.65 (1.32–2.06)
Sex				
Female	4,048	232	1.0 (ref)	1.0 (ref)
Male	3,995	205	1.10 (0.91–1.33)	1.06 (0.87–1.28)
Primary diagnosis				
Primary OA	6,152	309	1.0 (ref)	1.0 (ref)
Inflammatory disease	421	34	1.19 (0.83–1.69)	0.80 (0.55–1.15)
Fracture	266	7	0.84 (0.40–1.78)	0.71 (0.34–1.51)
Pediatric hip disease	722	53	1.96 (1.47–2.63)	1.57 (1.15–2.13)
Idiopathic femoral head necrosis	249	13	1.23 (0.71–2.14)	1.06 (0.61–1.86)
Secondary posttraumatic OA	39	5	1.83 (0.76–4.43)	1.40 (0.57–3.41)
Tumor	7	0	0.00 (0.00–∞)	0.00 (0.00–∞)
Other	52	8	1.13 (0.56–2.28)	0.90 (0.44–1.82)
Age				
0–49	1,992	179	1.0 (ref)	1.0 (ref)
50–59	3,147	173	0.59 (0.48–0.73)	0.59 (0.47–0.74)
60–75	2,677	82	0.42 (0.32–0.54)	0.42 (0.31–0.56)
> 75	227	3	0.54 (0.17–1.69)	0.61 (0.19–1.92)
Cup design				
Trilogy	5,536	70	1.0 (ref)	1.0 (ref)
Romanus	1,531	213	1.93 (1.45–2.56)	2.62 (1.90–3.63)
Harris-Galante	976	154	1.86 (1.37–2.51)	2.44 (1.77–3.37)
Stem fixation				
Cemented stem	4,414	290	1.0 (ref)	1.0 (ref)
Uncemented stem	3,618	147	1.11 (0.91–1.35)	0.76 (0.61–0.95)
^a^ ref: reference group.				
A Cox regression analysis was performed where covariates (age, sex, primary diagnosis, HA coating, cup design, and stem fixation) were initially entered as singular variables, and a crude risk ratio was calculated for each variable. Thereafter, all covariates mentioned above were entered in the regression model and risk ratios were mutually adjusted for all covariates. Crude and adjusted risk ratios (RRs) were calculated for revision due to aseptic loosening.				

**Table 5.** Relative risk (RR) of cup revision for any reason. 8,043 hips in 6,646 patients.

Endpoint: any reason	No. of patients	No. of revisions	Crude RR (95% CI)	Adjusted RR (95% CI)
Coating				
– HA	2,830	331	1.0 (ref) ^a^	1.0 (ref)
+ HA	5,213	245	1.11 (0.93–1.32)	1.44 (1.18–1.75)
Sex				
Female	4,048	265	1.0 (ref)	1.0 (ref)
Male	3,995	311	1.14 (0.97–1.35)	1.12 (0.94–1.32)
Primary diagnosis				
Primary OA	6,152	410	1.0 (ref)	1.0 (ref)
Inflammatory disease	421	42	1.13 (0.83–1.56)	0.80 (0.57–1.11)
Fracture	266	12	1.03 (0.58–1.83)	0.91 (0.51–1.62)
Pediatric hip disease	722	65	1.75 (1.34–2.27)	1.42 (1.08–1.87)
Idiopathic femoral head necrosis	249	17	1.19 (0.73–1.93)	1.06 (0.65–1.73)
Secondary posttraumatic OA	39	8	2.28 (1.13–4.59)	1.78 (0.87–3.61)
Tumor	7	1	6.29 (0.88–∞)	4.77 (0.67–∞)
Other	52	10	1.14 (0.61–2.14)	0.93 (0.50–1.76)
Age				
0–49	1,992	222	1.0 (ref)	1.0 (ref)
50–59	3,147	232	0.64 (0.53–0.77)	0.65 (0.53–0.79)
60–75	2,677	117	0.47 (0.38–0.59)	0.47 (0.37–0.60)
> 75	227	5	0.58 (0.24–1.41)	0.64 (0.26–1.57)
Cup design				
Trilogy	5,536	123	1.0 (ref)	1.0 (ref)
Romanus	1,531	254	1.69 (1.34–2.13)	2.04 (1.57–3.66)
Harris-Galante	976	199	1.78 (1.39–2.28)	2.16 (1.66–2.82)
Stem fixation				
Cemented stem	4,414	377	1.0 (ref)	1.0 (ref)
Uncemented stem	3,618	199	1.09 (0.92–1.29)	0.84 (0.69–1.02)
^a^ ref: reference group.				
A Cox regression analysis was performed where covariates (age, sex, primary diagnosis, HA coating, cup design, and stem fixation) were initially entered as singular variables, and a crude risk ratio was calculated for each variable. Thereafter, all covariates mentioned above were entered in the regression model and risk ratios were mutually adjusted for all covariates. Crude and adjusted risk ratios (RRs) were calculated for revision for any reason.				

An initial analysis was performed in which all covariates mentioned above were entered as singular variables, and a crude risk ratio was calculated for each variable. Thereafter, all of these covariates were entered in the regression model and risk ratios were mutually adjusted for all covariates. Crude and adjusted risk ratios were calculated both for revision due to aseptic loosening and for revision for any reason. All statistical analyses were performed using SPSS (version 16.0).

## Results

### Characteristics of the study population

The numbers of males and females were about equal. The largest number of THAs was found in the age groups between 50 and 75 years. Primary osteoarthritis was the most common preoperative diagnosis. The group with HA-coated cups was larger than the group with uncoated cups. By 2007, 685 (8.5%) of all 8,043 cups had been revised, mostly due to aseptic loosening (5.7%), dislocation (1.0%), or deep infection (0.8%).

### Risk of cup revision due to aseptic loosening

The crude risk ratio of HA coating for the risk of revision for aseptic cup loosening was 1.1 (95% CI: 0.98–1.5) without adjusting for covariates (Table 4). In the next step, the risk of revision due to aseptic cup loosening including all factors mentioned above as covariates was analyzed, and risk ratios of each covariate mutually adjusted for all other factors were calculated. We found that HA coating was a risk factor for cup revision due to aseptic loosening with an adjusted risk ratio of 1.7 (95% CI: 1.3–2.1). In this analysis age at primary arthroplasty of below 50 years, a diagnosis of previous pediatric hip disease, and the presence of Romanus or Harris-Galante cups were also associated with statistically significant increased risk of cup revision due to aseptic loosening. Use of a cemented stem was another risk factor (Table 4).

The type of hospital at primary arthroplasty was included in the Cox regression model, but was found not to statistically significantly influence the adjusted risk ratios of the covariates mentioned above (data not shown).

### Risk of cup revision for any reason

The crude risk ratio of HA coating for the risk of cup revision for any reason without adjustment for covariates was 1.1 (95% CI: 0.93–1.3) (Table 5). Subsequently, risk ratios of each covariate mutually adjusted for all other factors were calculated. The results were similar to those found in the analysis for the risk of revision due to aseptic loosening: HA coating was a risk factor for cup revision for any reason, with an adjusted risk ratio of 1.4 (95% CI: 1.2–1.8). The covariates age at primary arthroplasty below 50 years, the use of a cemented stem, and the presence of Romanus or Harris-Galant cups statistically significantly increased the risk of cup revision for any reason (Table 5).

The risk of revision due to infection was not influenced by the presence of an HA coating (data not shown).

## Discussion

Our analysis indicates that HA coating of acetabular cups influences the outcome. However, contrary to the results of some previous studies and a commonly expressed opinion (Moilanen et al. 1996, Roffman and Kligman 1999, Ali and Kumar 2003), cups with an HA coating did not perform better than identical cups without this surface finish (Röhrl et al. 2004). This finding is only in partial agreement with a large Danish register analysis of HA-coated hip implants that found no reduced risk of revision with use of HA-coated cups (Paulsen et al. 2007). In that study, the adjusted risk ratio of HA-coated cups was calculated to be 0.85 when compared to uncoated cups, but with a 95% CI ranging from 0.68 to 1.1. One possible reason for this discrepancy could be that a substantial proportion of the implants investigated in that study were Mallory-Head cups, a cup type that was not investigated in our study.

The cup design seems to be an important risk factor for revision. The Cox regression analysis showed that the Trilogy cup had a statistically significantly better survival than the Harris-Galante and the Romanus cups. This finding was not unexpected, as inferior results of the Romanus and the Harris-Galante cups have been reported previously (Thanner et al. 1999, Lyback et al. 2004, Hallan et al. 2006, Swedish Hip Arthroplasty Register 2007). In Harris-Galante cups, excessive liner wear was probably due to an insufficient locking mechanism, leading to “silent osteolyses” (Röhrl et al. 2006). It has also been described that threaded cups with HA coating perform better than some designs of hemispherical press-fit cups with HA coating, indicating that differences in fixation principles influence cup survival more than HA coating (Reikeras and Gunderson 2006). The main problems with the press-fit cups analyzed in that study were wear and a high frequency of thin liners. In our study, the cup size and liner thickness are not known, because this was not recorded in the registry database until 1999 and onwards. We do, however, have no reason to believe that the size of cups inserted with and without HA coating should have varied during the period studied, even if this type of bias cannot be completely ruled out. Finally, our study only embraced 3 designs. The only one of these still in general use does not seem to be significantly affected by the use of a ceramic coating (Trilogy).

The association of HA coated cups with stems of inferior performance could distort cup survival, as stem revision could in some cases have been combined with cup or at least liner revision “en passant”, without actual cup loosening being present. Analysis of the various combinations of cup and stem components in our study population does not suggest that this was the case (Table 6). In the group of Romanus cups coated with HA, 60% of patients received an HA-coated Bimetric stem. This stem has shown excellent survivorship in other studies (Lyback et al. 2004, Eskelinen et al. 2006). In the group of HA-coated Trilogy cups, the Spectron and Lubinus SPII stems represented between 20% and 30% each. None of these stems has been reported to show poor survival, and the Link SPII stem is considered one of the best-performing cemented stems in the registry (Aldinger et al. 2003, Swedish Hip Arthroplasty Register 2007). The Spectron EF predominated in the group of HA-coated Harris-Galante cups (42%), and this stem has a good performance (Swedish Hip Arthroplasty Register 2007).

**Table 6.** Distribution of stems combined with the cup types Romanus (A), Trilogy (B), and Harris-Galante (C)

	+ HA		– HA	
	n	%	n	%
A. Romanus Cup				
Bimetric + HA	262	60	141	13
Bimetric – HA	67	15	251	23
Bimetric cemented	15	3	359	33
Others	96	22	340	31
	440	100	1,091	100
B. Trilogy Cup				
Spectron EF primary	1,215	27	28	3
Lubinus SPII	1,026	23	70	7
CLS	942	21	479	46
Others	1,318	29	458	44
	4,501	100	1,035	100
C. Harris-Galante Cup				
Spectron EF	114	42	172	24
Lubinus SPII	26	10	245	35
Charnley	14	5	144	21
Others	118	43	143	20
	272	100	704	100

The degree to which the stem component contributes to cup survival is uncertain. In the Cox regression analysis, we found that the presence of a cemented stem is a risk factor for cup revision due to aseptic loosening when adjusted for all other covariates. This indicates that the stem component was an independent covariate with influence on outcome, despite the fact that the endpoint of our analysis was cup revision, either in the presence or absence of stem revision, whereas events consisting of stem revision alone were excluded. It should be noted that the registered indication for revision surgery was “aseptic loosening” of either the cup or both the cup and the stem, regardless of the underlying problem. In such cases, a revision of the cup or the liner was performed. The cohort of 8,043 hips we analyzed, with its wide array of different stem types and the high number of different cup-and-stem combinations, was not large enough for a comprehensive analysis of the factor “type of stem”. Moreover, due to the large number of degrees of freedom obtained if individual stem types are entered, a Cox regression model including this variable is not feasible. We could, however, rule out the possibility that HA cups were predominantly used in combination with stems of inferior performance.

It has been reported previously that younger patients have an inferior outcome after arthroplasty with HA-coated cup components (Manley et al. 1998). In this group of patients, hip arthroplasty survival is often limited by revision due to osteolysis, cup loosening, and/or excessive liner wear (Puolakka et al. 1999, Wangen et al. 2008). It could be that the HA coating in some designs, due to increased burden of released particles from the coating, facilitates these events. Detailed analysis of our revision cases including preoperative radiographs would be necessary to examine this issue further.

Several other covariates with a possible influence on cup survival were also investigated. The type of hospital at primary arthroplasty had no statistically significant influence on the adjusted risk ratios of the covariates entered in the Cox regression model. Some other possible confounding factors such as medication with steroids, non-steroidal anti-inflammatory drugs, or bisphosphonates that are known to influence bone metabolism were not recorded. The same applies to medical conditions that have no known direct effect on implant survival, but that could exert an indirect influence, e.g. overweight, diabetes mellitus, or lipid metabolism disorders.

We had hypothesized that HA coating would influence cup survival, expecting no or beneficial effects. In contrast to these expectations, HA coating had a negative effect on cup survival, with most marked effects in 1 of the 3 cup designs investigated (Romanus). Many of these cups were initially used with femoral heads made of titanium alloy. At the time period for their use, this parameter was not recorded. Thus, whether or not these heads were used more frequently with HA-coated cups is not known. Articulations with different head sizes (22, 28, or 32 mm) were used in the study population, but this parameter was not systematically registered until 1999, and was therefore not analyzed.

The HA coatings used in the 3 cup designs studied by us have shown high biological activity and fixation to bone in several studies, where release of calcium ions to the tissues close to the implant/bone interface has been regarded as an important initiator of bony ingrowth. It could be speculated that resorption of the HA coating occurs before secure bone ingrowth into the cup surface has been achieved. This could lead to inferior stability or loss of bone mineral density (BMD) when compared to uncoated cups, where long-lasting bone ingrowth can take place. Such loss of BMD has been described in the vicinity of HA-coated Trilogy cups (Digas et al. 2006). A retrieval analysis of HA-coated cups has shown degradation of HA in all retrieved cups, and the authors concluded that “[…] poor replacement of HA by bone may interfere with long-term fixation” (Rokkum et al. 2003). The hypothesis of generally inferior fixation of HA-coated cups is, however, contradicted by observations on reduced frequency of radiolucent lines around HA-coated cups (Moilanen et al. 1996) and a similar BMD around HA-coated and uncoated Trilogy or Cambridge cups (Field et al. 2006, Laursen et al. 2007). Abrasion of HA particles from the coating has been shown to lead to increased wear of the polyethylene liner, thus leading to periprosthetic osteolysis and early loosening (Morscher et al. 1998). This mechanism may be responsible for the inferior survival of HA-coated cups seen in our study.

In conclusion, our results derived from registry data on 8,043 hips indicate that HA coating does not enhance the survival of cups when using revision for aseptic cup loosening as an endpoint. On the contrary, with at least some designs, HA coating seems to be a risk factor for cup revision both due to aseptic loosening and for any reason, when adjusting for other covariates. In contrast, the risk of revision due to deep infection is not influenced by the type of cup coating. It should be emphasized that our study only embraces 3 cup designs that are available with or without HA coating. Because numerous other cups with pure HA or similar coatings are available on the market, further studies of other designs are mandatory. Until such information is available, we cannot generally recommend HA coating of the acetabular component in primary arthroplasty. The extra economic burden of HA coating added onto the surface of these cups does not seem to be justified.
